# Supplementary figures and images for: Identification of Southeast Asian Anopheles mosquito species using MALDI-TOF mass spectrometry
Source: PLoS One. 2024 Jul 5;19(7):e0305167. doi: 10.1371/journal.pone.0305167 (PMC11226003; doi:10.1371/journal.pone.0305167)

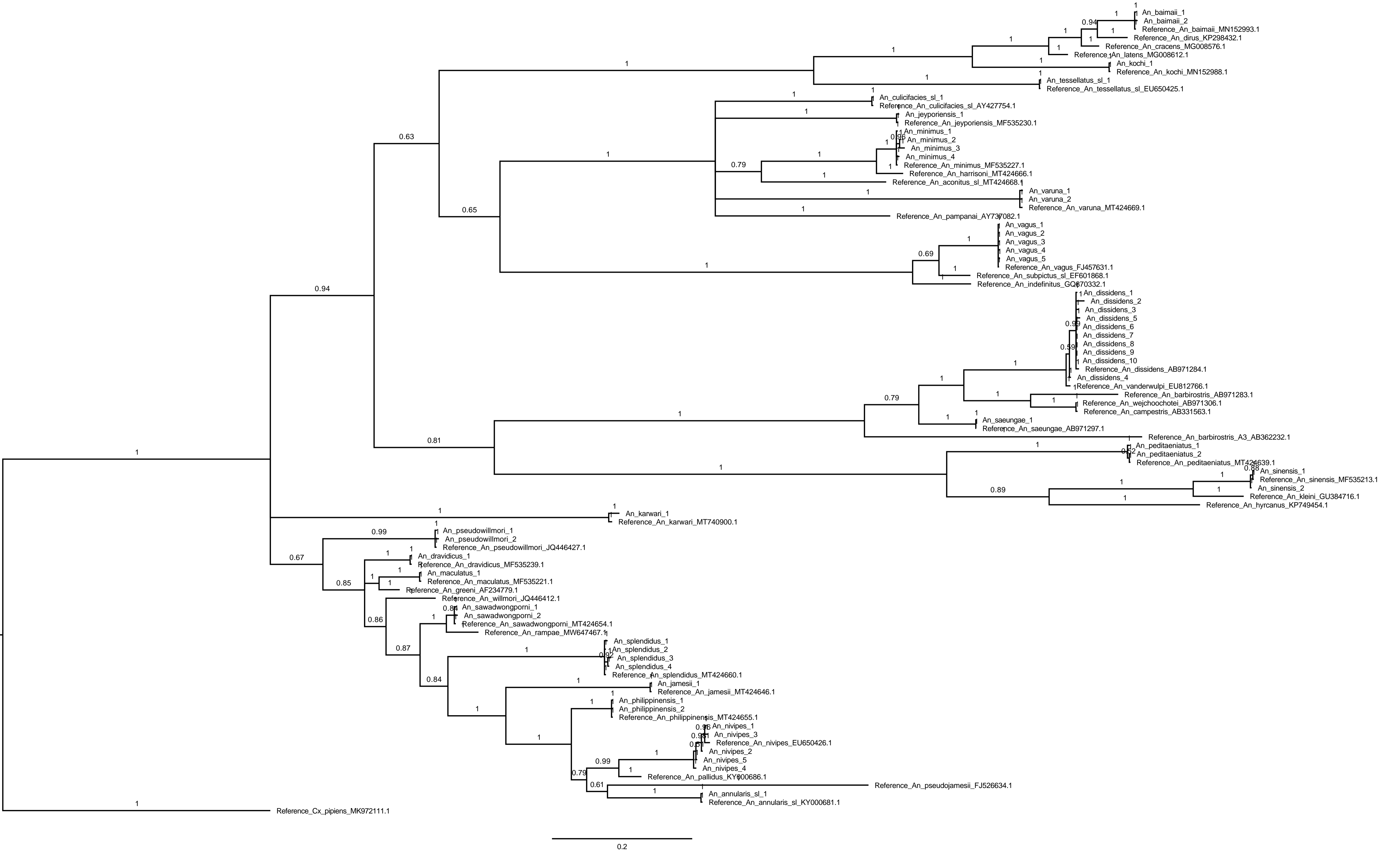

Supplement: S1 Fig — The tree is rooted on Culex pipiens. Branch are labeled with Bayesian posterior probabilities. The bar represents 0.2 substitutions per site. (PDF) [file pone.0305167.s001.pdf]

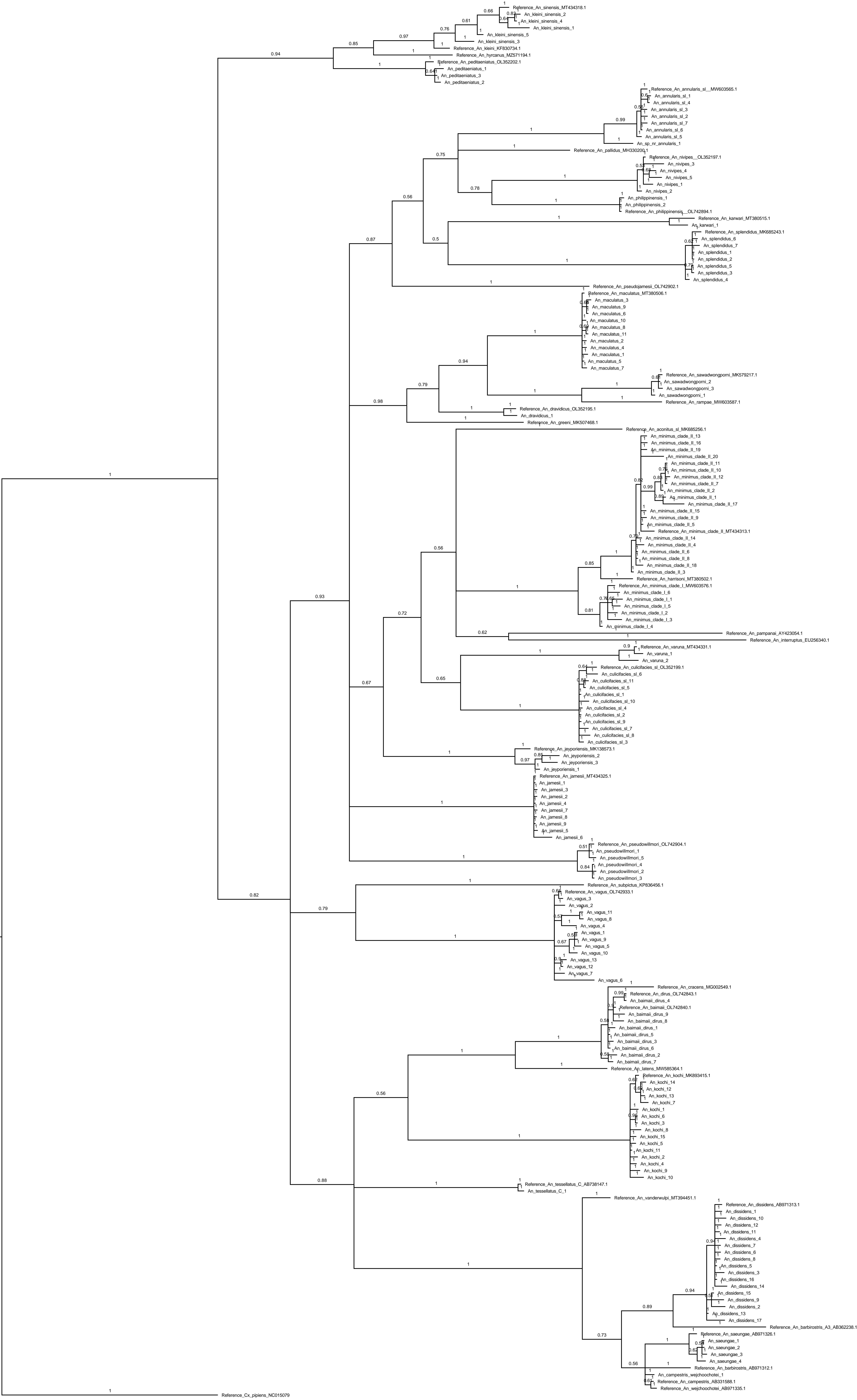

Supplement: S2 Fig — The tree is rooted on Culex pipiens. Branch are labeled with Bayesian posterior probabilities. The bar represents 0.02 substitutions per site. (PDF) [file pone.0305167.s002.pdf]
